# The effects of Astragalus, Epimedium, and Fructus Ligustri Lucidi extract on the antioxidant capacity, and immune status of goslings under stress conditions

**DOI:** 10.3389/fvets.2025.1558440

**Published:** 2025-06-05

**Authors:** Qing Qu, Yihong Sun, Yayan Liang, Yongsheng Nie, Ming Gao, Yaohui Yuan, Wei Wang, Shuo Zhou

**Affiliations:** ^1^Innovative Institute of Animal Healthy Breeding, Zhongkai University of Agriculture and Engineering, Guangzhou, China; ^2^College of Animal Science and Technology, Zhongkai University of Agriculture and Engineering, Guangzhou, China; ^3^Guangdong Laboratory for Lingnan Modern Agriculture, Guangzhou, China; ^4^Crown Bioscience (Zhongshan) Co., Ltd., Zhongshan, China; ^5^Shenyang Weijia Biotechnology Co., Ltd., Shenyang, China

**Keywords:** geese, stress, growth performance, inflammation, immune function

## Abstract

With the development and improvement of the scale, intensification, and production level of the goose farming industry, oxidative stress often occurs during the process of producing meat geese. This study employed the intraperitoneal injection method with a concentration of 2.96 mmol/mL H_2_O_2_ to establish a model of oxidative stress in goslings. The effects of Astragalus, Epimedium, and Fructus Ligustri Lucidi (AEF) extract on the immune function, antioxidant capacity, meat quality, intestinal morphology, and intestinal barrier integrity of goslings were investigated. Twenty-four 1-month-old Magang geese from the same batch were randomly divided into four groups: control group (C group), AEF treatment group (AEF group), H_2_O_2_ stress group (S group), AEF and H_2_O_2_ stress group (S + AEF group). The experiment lasted for 30 days, during which the AEF group and the S + AEF group were fed 0.1 g/mL of AEF aqueous solution, once daily. Hydrogen peroxide (H_2_O_2_) was employed as the stressor. On the 25th day of the experiment, the goslings were weighed and administered an abdominal cavity injection of H2O2. The results showed that compared with the control group, the body weight, spleen weight, bursa index of fabricius, spleen index, thymus index, the activity of glutathione peroxidase (GSH-PX), total superoxide dismutase (T-SOD), and catalase (CAT) in serum, liver, and jejunum were decreased in S group (*p* < 0.05). Meanwhile, compared with the S group, the body weight, spleen weight, bursa index of fabricius, spleen index, thymus index, the activity of glutathione peroxidase (GSH-PX), total superoxide dismutase (T-SOD), and catalase (CAT) in serum, liver, and jejunum were enhanced in S + AEF group and S + AEF + LP group (*p* < 0.05). However, the content of cortisol (CORT) and blood glucose were increased in S group (*p* < 0.05), AEF and AEF + LP effectively alleviated these growth trend. Supplementing with AEF and LP post-stress alleviated stress-induced the expression of inflammatory factors (TGF-*β*, TNF-*α*, IL-1β, IL-6), and improve L*, a*, b*, Ph45 (min), Ph24 (h) and drip loss in pectoral (*p* < 0.05). The intestinal morphology results showed that the villus structures in the duodenum and jejunum of goslings were short and sparse under stress conditions, with a significant decrease in the villus height/crypt depth ratio (Vh/Cd). After AEF treatments, the villus height increased, leading to an improved Vh/Cd in the small intestine. In conclusion, AEF can enhance the immune performance, and antioxidant capacity of goslings, thereby improving intestinal health. These findings offer new insights into enhancing immune performance and preventing stress in goslings.

## Graphical abstract

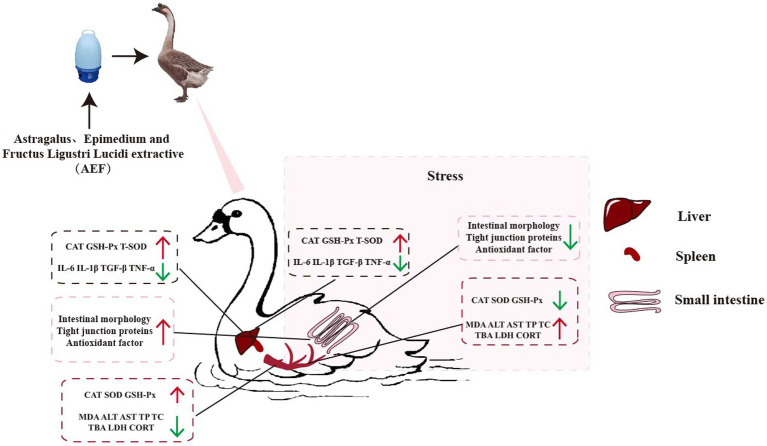

## Introduction

Geese in the modern poultry business are subjected to prolonged periods of intense pressure, high nutrition, and fast growth because to the rapid advancement of high-density farming practices. Stress factors have a significant impact on the antioxidant function and health of geese (1). Existing literature has reported that oxidative stress can have adverse effects on the health of geese. Oxidative stress depletes poultry antioxidant defenses, elevates free radicals, thereby compromising disease resistance and accelerating senescence or mortality (2). However, there are many factors that can cause stress in animals, including transportation, restraint, density, temperature variations, and the presence of loud and aggressive animals (3).

Existing literature has reported that H_2_O_2_ can induce oxidative damage by increasing the levels of reactive oxygen species (ROS) in cells and organisms (4, 5). Therefore, in this experiment, H_2_O_2_ was directly administered to the animal body to elevate the levels of reactive oxygen species (ROS) and establish an oxidative stress model in geese. H_2_O_2_ has been confirmed to cause damage to the intestinal epithelial barrier, reduce the immune levels of the organism, and induce oxidative damage (6, 7). However, after the onset of disease, farms prefer to use antibiotics to treat and control diseases (8). Prolonged use of antibiotics can lead to bacterial resistance, resulting in the emergence of “superbugs,” causing a decline in animal immune function, an increase in mortality, and other consequences (9). However, there are also some other commonly used anti-stress drugs. Due to stress being an extremely complex physiological process, these drugs are easily oxidized by the body during their action, rendering them ineffective. Therefore, finding a safe and effective therapeutic drug is urgent. Current literature reports that the use of probiotics and plant extracts can improve the intestinal microbial community of poultry, maintain intestinal health. The intestinal microbial community has been designated as a hidden metabolic “organ” because of its significant impact on host metabolism, physiology, nutrition, and immune function (10, 11). Plant extracts can effectively enhance human resistance, and they have promising prospects. As the demand for alternatives to antibiotics has become crucial, plant extracts are currently a viable option to partially replace antibiotics (12, 13). The interest in plant extracts is due to their natural properties, environmental friendliness, safety, and characteristics such as no drug residues, stable drugs, and minimal adverse reactions. They can improve animal immunity, increase egg production, and prevent and treat stress (14). In addition, probiotics are also an excellent choice for antibiotic replacement and are beneficial microbial strains for health. The animal gut is a complex ecosystem of microorganisms, host cells, etc. Probiotics regulate the immune system to prevent diseases in both humans and animals by interacting with the gut microbiota (15).

Astragalus, with its main components being polysaccharides, flavonoids, saponins, etc., is a commonly used traditional Chinese medicine in many herbal formulations. It is widely applied in clinical settings for its antioxidative, anti-inflammatory, anti-apoptotic properties, enhancing immunity, and alleviating aging (16, 17). As a green feed additive, Astragalus is utilized as a supplement for live microbial feed, improving intestinal barrier, maintaining microbial balance, and promoting gut health (18).

Epimedium is an herbal plant rich in flavonoid compounds, offering various beneficial effects to animals. Epimedium improves the composition of the gut microbiota by modulating the core genus Lactobacillus, thereby enhancing antioxidant capacity, intestinal permeability, and strengthening tight junction proteins (19).

*Ligustrum lucidum*, as a high-quality feed additive, can reduce the mortality of animals, cholesterol in cracked eggs, low-density lipoprotein cholesterol, triglycerides, and aspartate aminotransferase. Additionally, it increases the rate of high-density lipoprotein in serum and the cholesterol function in serum levels (20).

*Lactobacillus plantarum* is a kind of lactic acid bacterium that does not produce gas. *Lactobacillus plantarum* has long been employed for fermentation in vegetables, meat, and dairy products, but its significance as a probiotic is now being more widely acknowledged. It plays a crucial role in intestinal health, immune function, and brain health (21). Previous studies have demonstrated that feeding plant Lactobacillus to weaned piglets can enhance piglet growth performance, immune function, and regulate intestinal microbiota, among other significant effects (22).

A combination of Astragalus, Epimedium, and Fructus Ligustri Lucidi (AEF) with Plant Lactobacillus (LP) has the ability to boost the immune system of the host and promote the growth performance of animals. The therapeutic and preventative benefits of incorporating AEF into drinking water for pigeons. These benefits include increased stress tolerance, enhanced egg production, improved immunity, and better digestive health (19). Nevertheless, there is a scarcity of studies regarding the impact of incorporating AEF and AEF + LP into drinking water on stress resilience, antioxidant capability, immune response, and gastrointestinal well-being in goslings. Hence, we evaluated the effects of AEF and AEF + LP on the growth performance, antioxidant capacity, immunological function, and intestinal microbiota of goslings. This work establishes a theoretical foundation for utilizing AEF and AEF + LP to enhance the production performance and immunity of goslings. This experiment aims to investigate the impact of incorporating AEF (antioxidant-enriched feed) and AEF + LP (antioxidant-enriched feed combined with low protein) on the production performance of goslings. The evaluation will focus on assessing alterations in immunological and antioxidant capacity.

## Materials and methods

### Animals

Thirty-six 30-day-old goslings (from Guangdong Golden Livestock and Poultry Breeding Co., Ltd., China) were placed in a pathogen-free environment and raised freely. The goslings were randomly divided into 6 groups (6/group): Control group (C group), water supplemented with Astragalus, Epimedium, and Fructus Ligustri Lucidi extract (AEF) group, water supplemented with AEF and *Lactobacillus plantarum* (AEF + LP) group, stress group (S group), stress plus AEF group (S + AEF group), and stress plus AEF plus LP group (S + AEF + LP group) (Table 1).

**Table 1:** Experimental grouping and design for goslings.

Item	Add category	Give stimulation
C	Normal water	Nothing
AEF	Water with AEF	Nothing
S	Normal water	H_2_O_2_ stress
S + AEF	Water with AEF	H_2_O_2_ stress

### Preparation of hydrogen peroxide solution

Select a 30% hydrogen peroxide solution, perform gradient dilution with 0.75% saline, and calculate the final working concentration for use.

### Plant extracts and plant lactobacilli

Take 100 g of Astragalus, 60 g of Epimedium, 60 g of Ligustrum, 40 g of Chaihu, 40 g of Poria, and 30 g of Licorice. Decoct in water 10 times for 30 min. Combine the decoction, filter, concentrate the filtrate to about 330 mL, add 3 times ethanol, let it stand at 4°C for 20 h. Filter, recover ethanol from the filtrate, concentrate to a clear paste with a relative density of 1.25 to 1.35 (25°C). Add 3 parts of sugar and 1 part of starch according to the amount of clear paste, mix well, granulate, low-temperature drying, and then package separately. The concentration of AEF in water is 0.2 g/mL, 0.2 g per kilogram of goose body weight, gradually increasing with body weight, once a day.

The plant lactobacillus is purchased from Weikai Haisi (Shandong) Biotechnology Co., Ltd., administered to each goose once a day, with a dosage of 1 g per time.

### Animal treatment and tissue collection

The experiment lasted for 30 days, with AEF dissolved in physiological saline at 0.2 g/mL, LP dissolved in physiological saline at 1 g/mL, and geese treated with AEF, AEF + LP for 30 days. The basic diet used in the experiment was formulated in accordance with the Nutrient Requirements of Poultry (NRC) (1994) and that of the lion-head goose production industry in Guangdong Province. The composition and nutritional levels of the feed are listed in Table 2. In the last 5 days of the experiment, geese were subjected to H_2_O_2_ stress (H_2_O_2_ concentration of 2.96 mmol/kg). Geese had free access to feed and water. Blood was collected from the wing veins of each group of geese (*n* = 6) into blood collection tubes for serum preparation. 5 mL of blood was collected from the sub wing vein and placed at an angle until the serum was precipitated; subsequently, the blood was centrifuged at 3000 r/min for 15 min to separate the serum which was stored at −20°C for the determination of serum biochemical indexes and antioxidant indexes. After anesthesia, each group of 6 geese was euthanized, and the intestines were separated and rinsed with PBS solution. Tissues from the intestine, hypothalamus, liver, and spleen were used for real-time quantitative polymerase chain reaction (RT-qPCR) analysis. The intestines were fixed with a 4% paraformaldehyde solution and stained with HE for morphological observation.

**Table 2:** Composition of basal diets.

ltems	Content (%)
Ingredient	
Corn	58.00
Soybean meal	24.00
Bran	6.50
Rice bran	8.20
CaHPO4	1.20
Limestone	1.10
Premix^1^	1.00
Total	100.00
Nutrient levels^2^	
Metabolizable energy (M/kg)	11.50
Crude protein	17.46
Ether extract	4.07
Calcium	0.85
PhosphorusLysine	0.83
Metabolizable energy (M/kg)	0.89

1 The premix provided the following per kg of diets: 5000 [U, VB, 5.0 mg, VB, 8 mg, VB.5.0 mg, VB, 12 μg, VD, 800 1 U, VE 50 1 U, VK, 2.5 mg, folic acid 0.5 mg, nicotinamide 40mgbiotin 0.3 mg, pantothenic acid 25 mg, choline 1,500 mg, Fe (as ferrous sulfate) 85.2 mg, Cuas copper sulfate) 10 mg, Zn (as zinc sulfate) 50 mg, I (as potassium iodide) 0.3 mg, Se (as sodium selenite) 0.25 mg].

2 Metabolizable energy was a calculated value, while the others were measured values.

### Determination of serum indicators

Blood collected from the brachial vein of each group of geese was transferred to centrifuge tubes. Serum levels of Ca, alanine aminotransferase (ALT), alkaline phosphatase (ALP), aspartate aminotransferase (AST), total protein (TP), albumin‌ (ALB), GLB, A/G, triglyceride (TG), total cholesterol (TC), creatinine (CREA-S), UREA, creatine kinase (CK), total bilirubin (T-Bil-V), total bile acids (TBA), lactate dehydrogenase (LDH), and P were measured using the BS-240VET fully automated biochemical analyzer (Mindray Biomedical, Shenzhen). Goose serum CORT was assayed using an Elisa kit (Bolai Biotechnology Co., Ltd., Jiangsu, China). Specific procedures were performed following the manufacturer’s instructions.

### Morphological observations

All tissues were fixed in 4% paraformaldehyde at 4°C for 24 h, followed by processing and embedding in paraffin. Hematoxylin and eosin (H&E) staining was performed using standard protocols. Images of the slides were observed using a Leica microscope (DM500, Leica, Wetzlar, Germany). Villus height (Vh) and crypt depth (Cd) were measured using Image Pro Plus 6.0 software (Media Cybernetics, Rockville, MD).

### DNA extraction and sequencing

Collect fecal samples from each group of 5 geese for nucleic acid extraction. Following the manufacturer’s instructions, use the RNeasy Power Microbiome KIT (Qiagen, Milan, Italy) to extract total DNA. Amplify the gDNA of the V3-V4 region of the 16S rRNA gene to assess the microbial community (23). PCR products are tested on the Illumina MiSeq platform (Majorbio, Shanghai, China) according to the instructions (24).

### RT-qPCR analysis

Total RNA was extracted from the small intestine and liver using Trizol reagent (Invitrogen, Carlsbad, CA). The RNA concentration and quality were measured using a Q3000 Microspectrophotometer (Quawell Technology, Inc., Sunnyvale, CA). cDNA was synthesized using a commercial RT-PCR kit (Takara Shuzo Co., Ltd., Kyoto, Japan). The qPCR conditions were as follows: 95°C for 30 s; 40 cycles of 95°C for 5 s, 60°C for 34 s; 95°C for 15 s; 65°C for 5 s; 95°C for 5 s. Subsequently, real-time PCR was performed using the SYBR Green QuantiTect RT-PCR kit (Roche, South San Francisco, CA), and each sample was analyzed in triplicate. The 2^−ΔΔCt^ formula method was used to calculate elative fold-change values between samples. The primers used are provided in Table 3.

**Table 3:** Gene-special primers used in the real-time quantitative reverse-transcription PCR.

Gene	Primer (5′-3′)
β-Actin	Forward: GCACCCAGCACGATGAAAAT Reverse: GACAATGGAGGGTCCGGATT
IL-1β	Forward: CCGCTTCATCTTCTACCG Reverse: TGTAGGTGGCGATGTTGAC
IL-6	Forward: AAGTTGAGTCGCTGTGCT Reverse: GCTTTGTGAGGAGGGATT
TNF-α	Forward: GTTCTATGACCGCCCAGTTC Reverse: CACACGACAGCCAAGTCAA
TJP1	Forward: AGACCATTCCAGACATTCTCCACAG Reverse: CGCCTGCCACCTCTTCCATAG
Occluding	Forward: TGCTCTGCCTCATCTGCTTCTTC Reverse: CGTTCTTCACCCACTCCTCCAC
GSH-Px	Forward: ACAGATTAAGGCGTTTGCTGAGAAC Reverse: GGCTGCTCCTTCATCCACTTCC
SOD1	Forward: CATCTCTCTGACTGGACCACACTG Reverse: AGTTAGCGTGCTCTCGTTATCTCC
TGF-β	Forward: TCATCTCCATCTACAACAGCACCAG Reverse: GCATAATACTCCTCGTCGCTCCTC

### Determination of growth performance

During the growth performance assessment, fasting geese were weighed on the first and thirtieth days.

### Measurement of transpiration loss

Weigh muscles of similar size on the same side, place them in self-sealing bags, hang them in a refrigerator at 4 degrees overnight, and after removal, gently wipe the muscles with filter paper before weighing and calculating.

### Muscle mass assessment

Left pectoral muscle were measured. Measure the pH value and color of the muscles using a pH meter (Lunz Technology, Model 205) and a colorimeter (China Corporation, nr20xe) after 45 min and 24 h (stored at 4°C).

### Determination of the immunological organ index

Weighing of the thymus, spleen, and bursa of Fabricius during sampling (organ weight/body weight × 100%).

### Determination of antioxidant capacity

According to the instructions of the kit (Nanjing Jiancheng Bioengineering Institute), the activities of superoxide dismutase (SOD), catalase (CAT), and glutathione peroxidase (GSH-Px), as well as the content of malondialdehyde (MDA) and the total antioxidant capacity (T-AOC), were determined in serum, liver, and duodenum.

### Statistical analysis

Statistical analysis was conducted on table data using IBM SPSS Statistics V25.0 software (SPSS, Inc., Chicago, IL) through one-way analysis of variance (ANOVA). Differences between groups were determined by Duncan’s multiple range test. Data were presented as mean ±SD, and differences between treatments were considered statistically significant when *p* < 0.05 and highly significant when *p* < 0.01. Quantitative PCR was performed in triplicate, and representative results are shown. Statistical analysis was performed with Student’s *t*-test or one-way ANOVA, using GraphPad Prism 7.0 (GraphPad Software, San Diego, CA). A *p* value < 0.05 was considered statistically significant.

## Results

### Effects of AEF and AEF + LP on goose body weight, spleen weight, energy metabolism, serum antioxidant capacity, liver and intestinal antioxidant capacity under H_2_O_2_-induced stress

**Figure 1:**
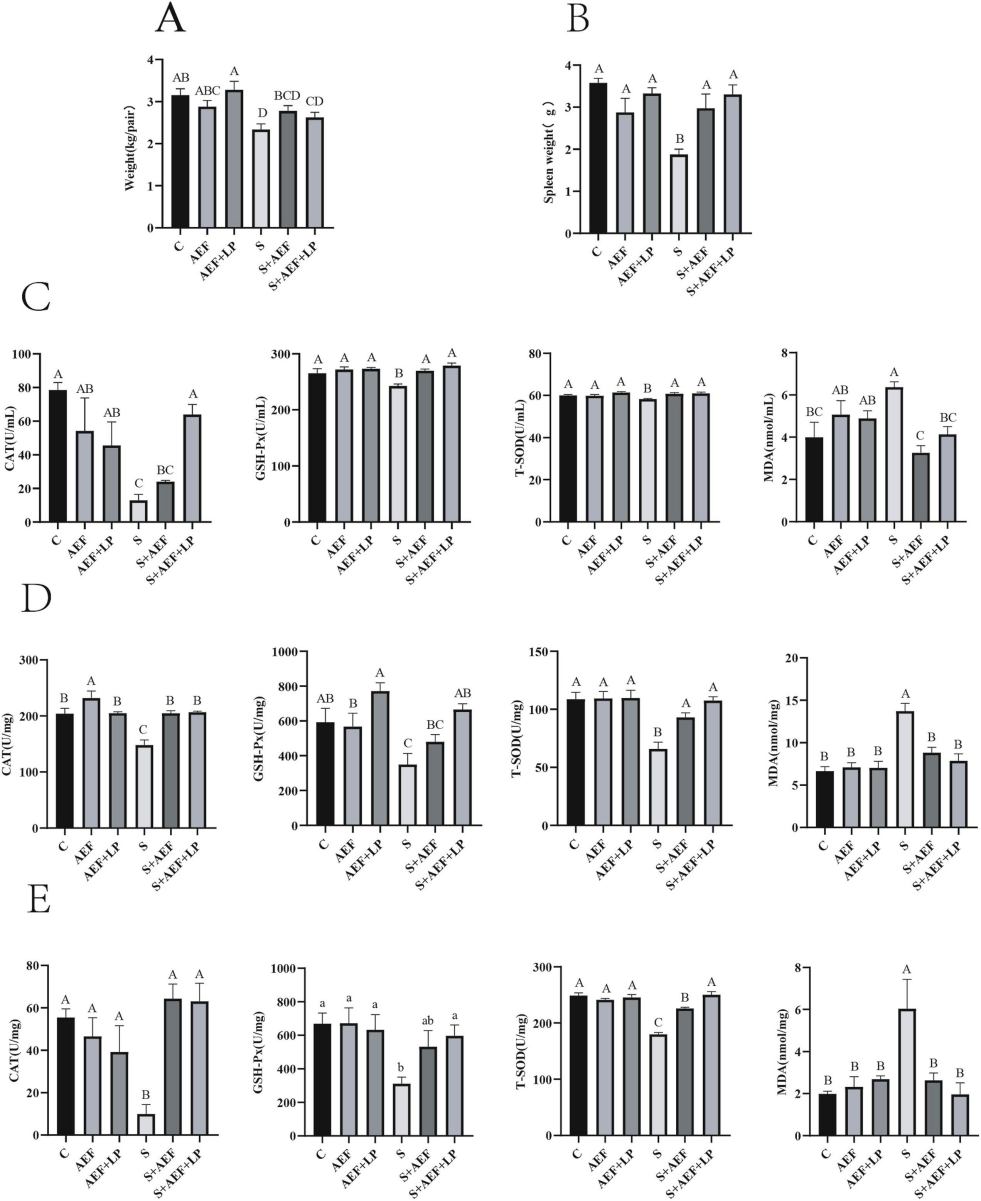
Impact of AEF and AEF + LP on the body weight and antioxidant capacity of goslings under H_2_O_2_ stress. **(A)** Gosling body weight reduction. Values are expressed as mean ± SEM (*n* = 4). **(B)** Gosling spleen weight reduction. Values are expressed as mean ± SEM (*n* = 4). **(C)** Impact of AEF and AEF + LP on the serum antioxidant enzyme activity in goslings. Values are expressed as mean ± SEM (*n* = 4). **(D)** Impact of AEF and AEF + LP on the liver antioxidant enzyme activity in goslings. Values are expressed as mean ± SEM (*n* = 4). **(E)** Impact of AEF and AEF + LP on the intestinal antioxidant enzyme activity in goslings. Values are expressed as mean ± SEM (*n* = 4). ^ab; ABCD^ In the same column, no letters or letters with the same superscript indicate no significant difference (*p* > 0.05), while letters with different lowercase superscripts indicate significant differences (*p* < 0.05), and letters with different uppercase superscripts indicate highly significant differences (*p* < 0.001).

As shown in Figure 1A, the stress group exhibited the greatest reduction in body weight. Compare with C group, the body weight was decreased in S group (*p* < 0.05). Furthermore, compared with the S group, the body weight was enhanced with AEF and LP treatment (*p* < 0.05). Subsequently, the weight of the spleen was measured. Stress-induced spleen atrophy was inhibited by AEF and AEF + LP treatments (*p* < 0.05) (Figure 1B). Serum antioxidant indicators were also detected by ELISA. Compared to the control group, the content of CAT, GSH-PX, and T-SOD in goose serum decreased (*p* < 0.05), while MDA increased under stress conditions (*p* < 0.05). AEF and AEF + LP treatments enhanced the antioxidant capacity of geese (*p* < 0.05) (Figure 1C). According to Figure 1D, compared to the control group, CAT, GSH-PX, and T-SOD content in the goose liver decreased, and MDA content increased under stress conditions (*p* < 0.05). AEF and AEF + LP treatments improved the antioxidant capacity of geese. In the comparison with the control group, CAT, GSH-PX, and T-SOD content in the goose intestine decreased, and MDA content increased under stress conditions (*p* < 0.05). AEF and AEF + LP treatments enhanced the antioxidant capacity of geese (*p* < 0.05) (Figure 1E). Serum biochemical indicators in geese were determined, showing that ALT, AST, TC, CREA-S, UREA, CK, TBA, LDH, and P levels increased after stress, while ALP, TP, ALB, GLB, A/G, and TG levels decreased. Metabolic capacity decline was relieved after AEF and AEF + LP treatments (*p* < 0.05) (Table 4).

**Table 4:** The effects of AEF on the serum biochemical parameters of goslings.

Item	C	AEF	S	S + AEF	SEM	*p*-value
Ca	2.45 ± 0.04	2.59 ± 0.24	2.22 ± 0.13	2.44 ± 0.17	0.038	0.063
ALT	17.67 ± 2.09^B^	16.63 ± 5.37^BC^	24.70 ± 5.17^A^	10.98 ± 1.88^D^	1.229	<0.001
ALP	920.67 ± 247.67^A^	754.58 ± 207.44^A^	391.35 ± 24.58^B^	322.43 ± 55.09^B^	52.697	<0.001
AST	13.40 ± 2.34^BC^	13.85 ± 2.35^BC^	24.53 ± 7.27^A^	8.50 ± 1.73^C^	1.295	<0.001
TP	56.85 ± 6.49^AB^	55.90 ± 5.41^AB^	49.38 ± 1.63^B^	63.68 ± 6.91^A^	1.354	0.008
ALB	15.85 ± 0.93^a^	15.85 ± 0.54^a^	12.82 ± 0.78^b^	15.20 ± 0.96^a^	0.366	0.044
GLB	41.57 ± 6.48^ab^	41.38 ± 8.28^ab^	35.25 ± 1.60^b^	49.07 ± 6.94^a^	1.476	0.013
A/G	0.43 ± 0.05^ab^	0.47 ± 0.05^a^	0.35 ± 0.06^b^	0.40 ± 0.00^ab^	0.014	0.031
TG	0.99 ± 0.14^A^	0.75 ± 0.07^B^	0.45 ± 0.06^C^	0.58 ± 0.11^BC^	0.041	<0.001
TC	3.79 ± 0.18^B^	3.99 ± 0.14^AB^	4.39 ± 0.18^A^	3.58 ± 0.46^B^	0.077	0.006
CREA-S	17.38 ± 1.87^B^	19.50 ± 2.51^B^	24.03 ± 3.38^A^	17.50 ± 1.30^B^	0.656	0.001
UREA	0.34 ± 0.12^bc^	0.40 ± 0.17^ab^	0.54 ± 0.13^a^	0.18 ± 0.05^c^	0.032	0.012
CK	548.50 ± 113.09^B^	659.30 ± 85.84^B^	903.17 ± 323.37^A^	277.32 ± 54.91^C^	52.105	<0.001
T-Bil-V	0.68 ± 0.24^a^	0.62 ± 0.13^a^	0.23 ± 0.18^b^	0.58 ± 0.15^a^	0.044	0.028
TBA	24.13 ± 6.36^B^	23.25 ± 11.81^B^	40.50 ± 6.98^A^	19.28 ± 4.94^B^	2.148	0.004
LDH	176.42 ± 28.06^C^	197.17 ± 34.72^BC^	289.25 ± 32.86^A^	154.15 ± 33.49^C^	11.561	<0.001
P	2.00 ± 0.42^ab^	2.07 ± 0.29^ab^	2.28 ± 0.13^a^	1.80 ± 0.08^b^	0.055	0.23

^abc^; ^ABCD^ In the same column, values without letters or with the same superscript letters indicate no significant difference (*p* > 0.05), while values with different lowercase superscript letters indicate significant differences (*p* < 0.05), and values with different uppercase superscript letters indicate extremely significant differences (*p* < 0.01). The values are presented as mean ± SD (*n* = 4). calcium (Ca), aminotransferase (ALT), alkaline phosphatase (ALP), aminotransferase (AST), total protein (TP), albumin (ALB), globulin (GLB), albumin/globulin (A/G), triglyceride (TG), total cholesterol (TC), creatinine (CREA-S), carbamide (UREA), creatine kinase (CK), total bilirubin (T-Bil-V), total bile acid (TBA), lactate dehydrogenase (LDH), and phosphorus (P).

### The impact of AEF and AEF + LP on corticosteroids and blood glucose in goslings under H_2_O_2_ stress

**Figure 2:**
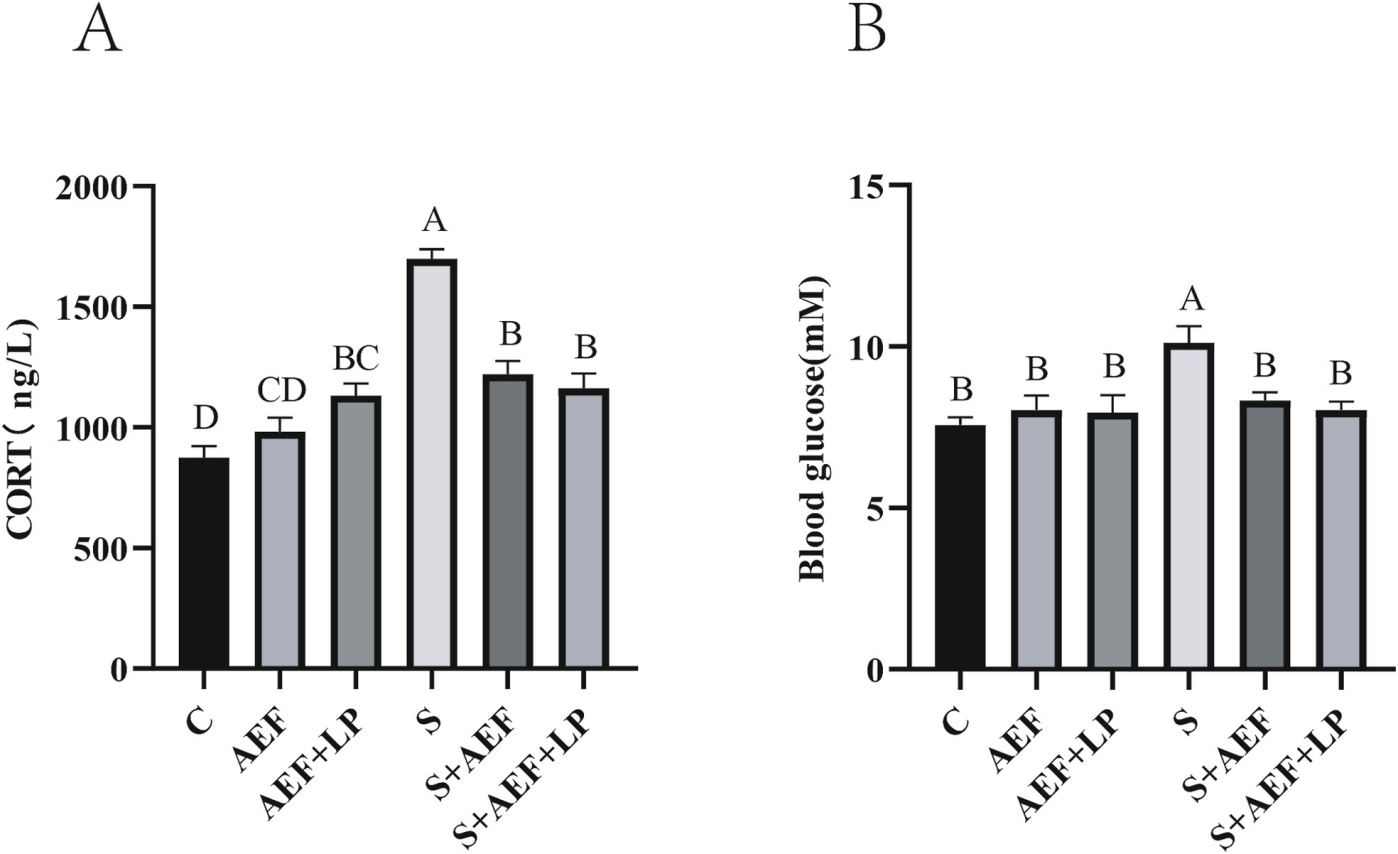
The effects of AEF and AEF + LP on corticosterone and blood glucose in goslings under H_2_O_2_ stress. **(A)** The content of corticosterone in gosling serum. Values are expressed as mean ± SEM (*n* = 4). **(B)** The content of blood glucose in gosling serum. Values are expressed as mean ± SEM (*n* = 4). The same letters or letter superscripts in the same column indicate no significant difference (*p* > 0.05), while different uppercase letter superscripts indicate extremely significant differences (*p* < 0.001).

Figure 2A demonstrates a considerable rise in CORT levels under stress situations as compared to the control group (*p* < 0.05). Treatment with AEF and AEF + LP effectively alleviated the content of CORT, and at the same time, AEF and AEF + LP reduced the elevation of blood glucose caused by stress (*p* < 0.05) (Figure 2B).

### The alleviating effect of AEF and AEF + LP on gosling intestine under H_2_O_2_ stress

**Figure 3:**
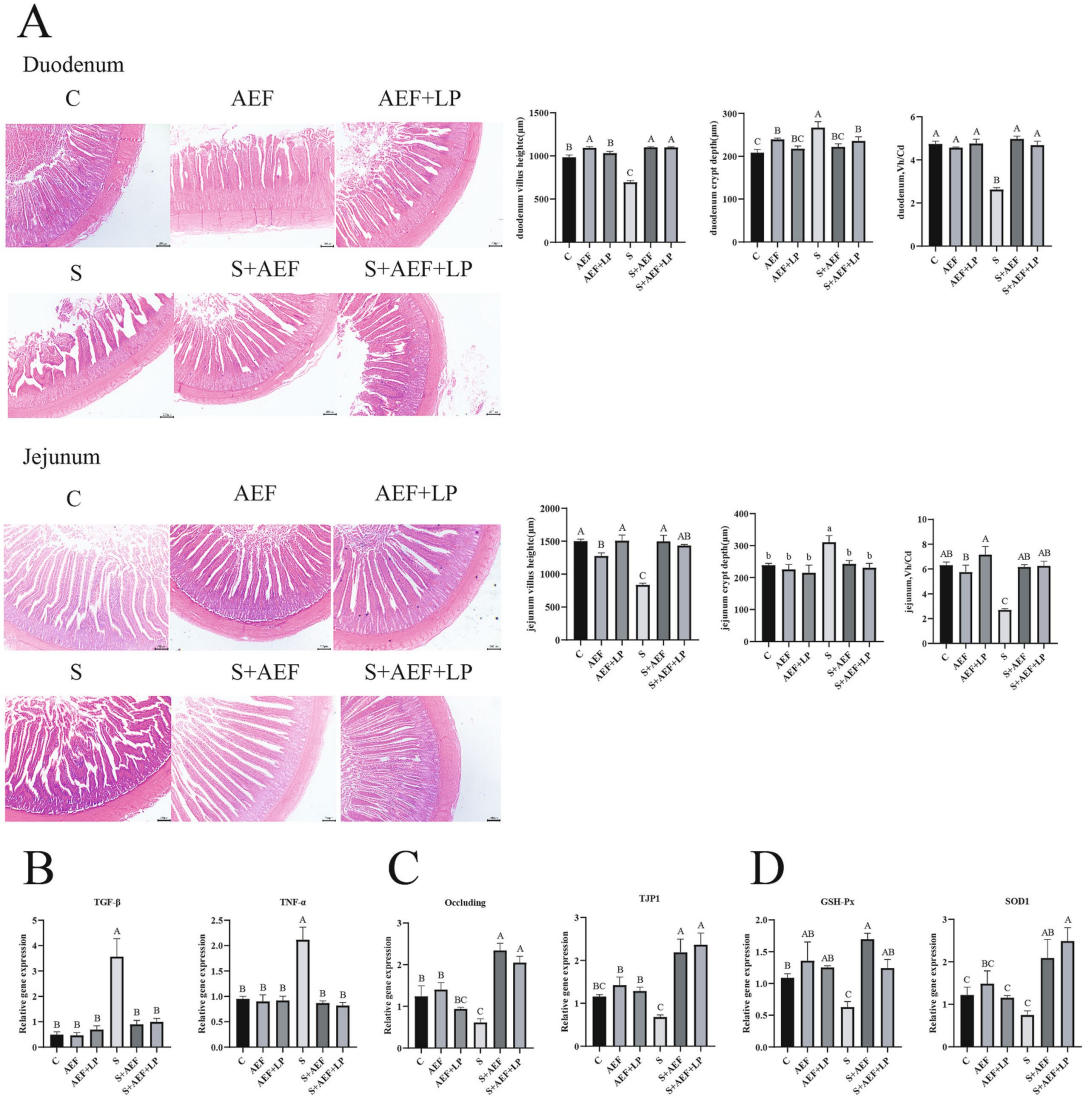
The impact of AEF and AEF + LP on the duodenum of goslings under H_2_O_2_ stress. **(A)** Typical morphology and Vh/Cd of the gosling duodenum. Scale: 200 mm. Values are presented as the mean ± SEM (*n* = 3). **(B)** RT-qPCR detection of mRNA expression levels of TGF-*β* and TNF-*α* in the jejunum. Values are presented as the mean ± SEM (*n* = 4). **(C)** RT-qPCR detection of mRNA expression levels of tight junction protein-related genes Occludin and TJP1 in the jejunum. Values are presented as the mean ± SEM (*n* = 4). **(D)** RT-qPCR detection of mRNA expression levels of antioxidant-related genes GSH-Px and SOD1 in the jejunum. Values are presented as the mean ± SEM (*n* = 4). ^ab; ABC^ In the same column indicate no significant difference (*p* > 0.05), while different lowercase letters indicate significant differences (*p* < 0.05), and different uppercase letters indicate extremely significant differences (*p* < 0.001).

Under stress conditions, the villous structure of the duodenum and jejunum in goslings becomes sparse and short. Statistical data on VH (villous height) and CD (crypt depth) in the duodenum and jejunum show a significant reduction in VH/CD in the small intestine under stress. Treatment with AEF and AEF + LP effectively improves VH, thus restoring the VH/CD ratio (*p* < 0.05) (Figure 3A). The expression of several inflammatory factor mRNAs was detected to assess the alleviating effect of AEF and AEF + LP on the jejunum under H_2_O_2_-induced stress. Compared to the control group, the mRNA expression levels of TGF-*β* and TNF-*α* in the jejunum significantly increased under stress, and their expression was reduced with the administration of AEF and AEF + LP (*p* < 0.05) (Figure 3B). Furthermore, RT-qPCR analysis revealed that, compared to the control group, AEF and AEF + LP significantly increased the expression of Occludin and TJP1 in the jejunum after stress treatment (*p* < 0.05) (Figure 3C). Finally, the expression of antioxidant genes in the jejunum was evaluated, and the administration of AEF and AEF + LP significantly enhanced the mRNA expression of GSH-Px and SOD1 after stress treatment (*p* < 0.05) (Figure 3D).

### The impact of AEF and AEF + LP on the gosling liver under H_2_O_2_ stress

**Figure 4:**
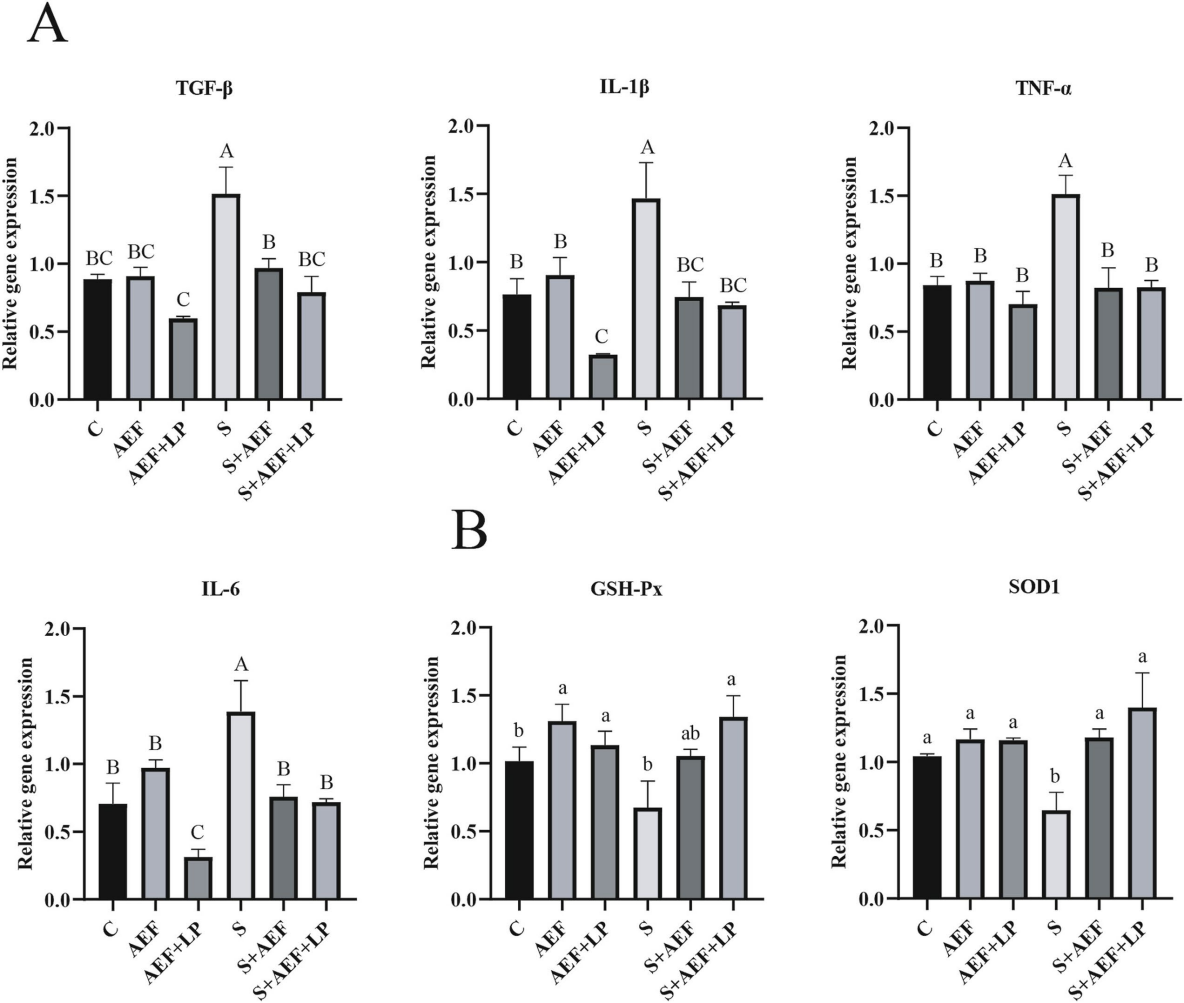
AEF and AEF + LP on the gosling liver under H_2_O_2_ stress. **(A)** The mRNA expression levels of TGF-β, IL-1β, TNF-α, and IL-6 in the liver were detected by RT-qPCR. Values are presented as the mean ± SEM (*n* = 4). **(B)** The mRNA expression levels of antioxidant enzyme-related genes GSH-Px and SOD1 in the liver were measured by RT-qPCR. Values are presented as the mean ± SEM (*n* = 4). ^ab; ABC^ In the same column, values with the same letter or superscript indicate no significant difference (*p* > 0.05), while values with different lowercase superscripts indicate significant differences (*p* < 0.05), and values with different uppercase superscripts indicate extremely significant differences (*p* < 0.001).

Under stress conditions, the liver undergoes certain damage. Therefore, we examined the expression levels of several common inflammatory factor mRNAs in the liver under stress conditions after administration of AEF and AEF + LP. Compared to the control group, the mRNA expression levels of TGF-*β*, IL-1β, TNF-*α*, and IL-6 were significantly increased under stress conditions, and AEF and AEF + LP had a certain mitigating effect on inflammation, reducing their expression levels (*p* < 0.05) (Figure 4A). At the same time, the expression of antioxidant genes in the liver was also measured. Administration of AEF and AEF + LP significantly increased the antioxidant capacity after stress (*p* < 0.05) (Figure 4B).

### The impact of AEF and AEF + LP on goose immune organ index, meat quality, and meat color under H_2_O_2_ stress

Compared to the control group, under stress conditions, the bursa of Fabricius index, spleen index, and thymus index of geese significantly decreased. Simultaneously, compared to the stress group, after AEF and AEF + LP treatment, the bursa of Fabricius index, spleen index, and thymus index showed a significant increase (*p* < 0.05) (Table 5). Compared to the control group, the L*, a*, and b* of geese showed no significant changes under stress conditions. However, drip loss and pH45 min significantly increased, while pH24 h decreased. In comparison with the stress group, after AEF and AEF + LP treatment, gosling’s drip loss significantly decreased, pH45 min significantly decreased, and pH24 h significantly increased (*p* < 0.05) (Table 6).

**Table 5:** The effects of AEF and AEF + LP on the immune indices of goslings.

Item	C	AEF	AEF + LP	S	S + AEF	S + AEF + LP	SEM	*p*-value
Bursa index of Fabricius (%)	0.09 ± 0.01^A^	0.09 ± 0.01^A^	0.09 ± 0.01^A^	0.05 ± 0.00^B^	0.08 ± 0.02^A^	0.08 ± 0.01^A^	0.004	<0.001
Spleen index (%)	0.13 ± 0.02^A^	0.11 ± 0.01^AB^	0.11 ± 0.01^B^	0.08 ± 0.01^C^	0.13 ± 0.01^AB^	0.12 ± 0.01^AB^	0.004	0.001
Thymus index (%)	0.14 ± 0.02^AB^	0.15 ± 0.02^A^	0.12 ± 0.01^BC^	0.08 ± 0.00^D^	0.11 ± 0.01^C^	0.10 ± 0.01^C^	0.006	<0.001

^ABCD^ In the same column, values without letters or with the same superscript letters indicate no significant difference (*p* > 0.05), while values with different lowercase superscript letters indicate significant differences (*p* < 0.05), and values with different uppercase superscript letters indicate extremely significant differences (*p* < 0.01). Values are presented as mean ± SD (*n* = 4).

**Table 6:** The influence of AEF and AEF + LP on goose meat quality.

Item	C	AEF	AEF + LP	S	S + AEF	S + AEF + LP	SEM	*p*-value
L*	37.84 ± 3.73	37.03 ± 3.23	38.11 ± 2.45	38.88 ± 1.55	38.27 ± 3.15	38.59 ± 5.53	0.643	0.981
a*	10.11 ± 2.85	10.50 ± 2.64	10.13 ± 3.26	10.18 ± 1.85	10.01 ± 1.77	10.09 ± 2.27	0.452	1
b*	7.15 ± 1.08	7.78 ± 1.24	7.00 ± 1.77	9.25 ± 2.15	7.65 ± 1.09	7.38 ± 1.66	0.32	0.396
Ph45 (min)	6.44 ± 0.08^B^	6.36 ± 0.30^B^	6.47 ± 0.18^B^	7.20 ± 0.25^A^	6.59 ± 0.16^B^	6.59 ± 0.24^B^	0.069	<0.001
Ph24 (h)	6.11 ± 0.14^A^	6.03 ± 0.04^A^	6.09 ± 0.05^A^	5.46 ± 0.34^B^	6.06 ± 0.14^A^	6.03 ± 0.07^A^	0.055	<0.001
Drip loss	1.41 ± 0.44^B^	1.17 ± 0.34^B^	0.78 ± 0.29^B^	2.23 ± 0.23^A^	1.13 ± 0.80^B^	0.74 ± 0.19^B^	0.13	0.001

^AB^ In the same column, values without letters or with the same superscript letters indicate no significant difference (*p* > 0.05), while values with different lowercase superscript letters indicate significant differences (*p* < 0.05), and values with different uppercase superscript letters indicate extremely significant differences (*p* < 0.01). Values are presented as mean ± SD (*n* = 4). *No practical significance, the meat quality detector displays L*.

### The impact of AEF and AEF + LP on the abundance of gosling cecal contents under H_2_O_2_ stress

**Figure 5:**
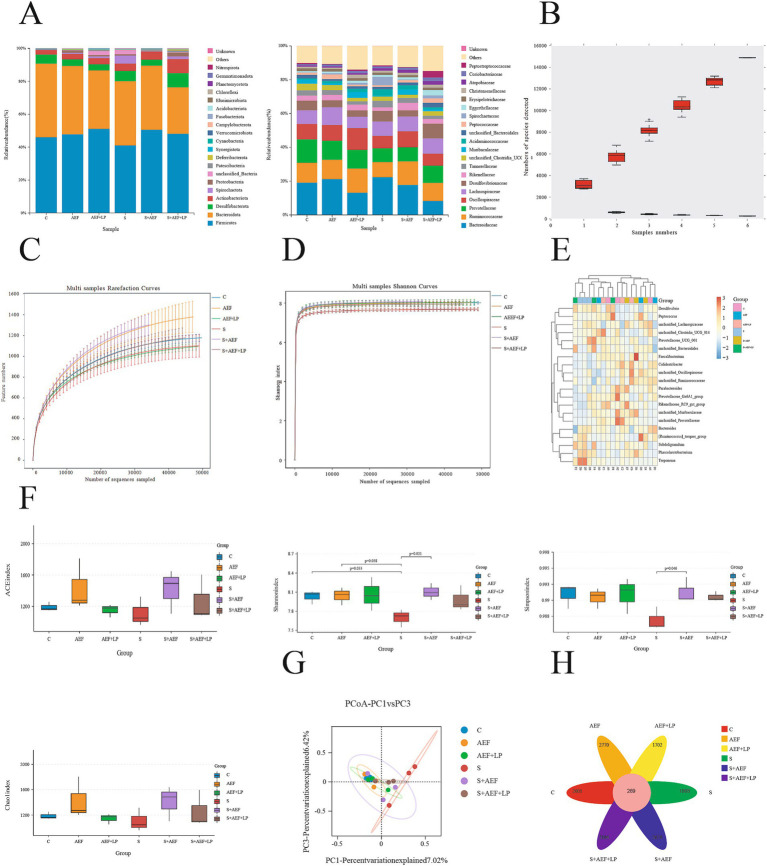
Abundance and diversity of cecal contents. **(A)** Relative abundance of 16S rRNA gene sequences at the phylum and family levels in gosling cecal contents (*n* = 3). **(B)** Species accumulation curve (*n* = 3). **(C)** Dilution curve (*n* = 3). **(D)** Shannon index curve (*n* = 3). **(E)** Heatmap of species composition (*n* = 3). **(F)** Alpha diversity indices (*n* = 3). **(G)** Principal coordinate analysis (PCoA) (*n* = 3). **(H)** Venn diagram (*n* = 3).

Detection of the microbial composition of gosling cecal contents using 16S rRNA Illumina MiSeq. In this study, 1,103,008 effective sequences were selected from 15 samples for subsequent analysis. Each sample had an average of 993 operational taxonomic units (OTUs). There were significant differences at the phylum and family levels among these six groups. The bar chart of phylum-level microbial structure showed that the relative abundance of Firmicutes in the six groups was 46, 47, 51, 41, 50, and 48%, respectively. At the family level, the bar chart of microbial structure showed that the relative abundance of Ruminococcaceae in the six groups was 11.7, 11.4, 14.3, 8.9, 13.9, and 11% (Figure 5A). When the sample size was sufficiently large in the species accumulation curve, the total number of species in the community would no longer significantly increase with an increase in sample size, and the curve would become flat (Figure 5B). In the dilution curve, a flat curve indicated a reasonable sample size (Figure 5C). As the sequencing depth increased, the Shannon index curve flattened, indicating that the sequencing depth was sufficient to reflect the majority of microbial information in the samples (Figure 5D). The relative abundance of 16S rRNA gene sequences in gosling cecal contents is shown, and the heatmap depicts the 20 most important bacterial genera in the contents (Figure 5E). Observing the species count, Simpson index, and Shannon index reflected the richness and evenness of species. Compared to the control group, Simpson and Shannon indices of goslings under stress showed a decreasing trend, indicating a decrease in species richness and abundance, which to some extent was restored after AEF and AEF + LP treatment (Figure 5F). Compared to the control group, there was a large degree of community dispersion among the samples in the S group, and this dispersion was somewhat reduced after the administration of AEF and AEF + LP (Figure 5G). We obtained 2001, 2,770, 1702, 1868, 2,676, and 2,104 samples from groups C, AEF, AEF + LP, and S, S + AEF, S + AEF + LP, respectively, with 269 OTUs shared among the six groups (Figure 5H).

## Discussion

The cattle sector has experienced significant growth in recent years, leading to the widespread adoption of intensive farming as the predominant agricultural strategy. Nevertheless, poultry and cattle are susceptible to stress responses in this agricultural approach, resulting in a decrease in animal output, compromised immunological function, and potentially fatal outcomes, hence resulting in substantial financial losses for farms. The hydrogen peroxide model is a classical model for studying immune stress in poultry and livestock (25). Previous studies have found that the negative effects of environmental changes directly impact the health and productivity of poultry, resulting in decreased feed intake, body weight, and immune function (26). However, oxidative stress also severely affects poultry production. Injection of hydrogen peroxide can lead to a decrease in poultry antioxidant capacity, feed-to-weight ratio, and muscle quality, indicating that hydrogen peroxide can directly impact the production performance and immune function of goslings (27). Prior research has indicated that extracts derived from Astragalus, Epimedium, and Ligustrum had therapeutic and preventative properties that enhance stress tolerance, egg production, immunology, and intestinal health in pigeons (28). Meanwhile, feeding weaned piglets with plant-derived Lactobacillus can enhance piglet growth performance, immune function, and regulate intestinal microbiota, among other critical effects (22). However, the therapeutic and preventive effects of the combined use of Astragalus, Epimedium, Ligustrum extracts, and plant-derived Lactobacillus on hydrogen peroxide stress in goslings remain unclear. Therefore, this experiment investigated the impact of Astragalus, Epimedium, Ligustrum extracts, as well as the combination of Astragalus, Epimedium, Ligustrum extracts, and plant-derived Lactobacillus on the immune and antioxidant capabilities of goslings. The results indicate that adding 0.2 g/mL AEF and 0.2 g/mL AEF + 1 g/mL LP per kilogram of gosling body weight to the water can enhance the goslings’ stress resistance. Thus, the addition of AEF and AEF + LP to drinking water can promote the growth of goslings and alleviate stress.

When the organism is influenced by external or internal stressors, oxidative stress is generated, leading to the destruction of prooxidants and antioxidants in cells. The reduced activity of antioxidant enzymes in the body results in DNA hydroxylation, protein denaturation, increased lipid peroxidation, and impairment of cellular functions, disrupting the conditions necessary for cell survival. Catalase (CAT), glutathione peroxidase (GSH-PX), and total superoxide dismutase (T-SOD) are important antioxidant markers in the body. In response to external or internal stress stimuli, the organism activates the antioxidant defense system to maintain the oxidative-reductive balance within cells and uphold internal homeostasis (29, 30). When the organism undergoes oxidative stress, MDA, as a biomarker of lipid and protein oxidation, directly reflects the degree of lipid damage in the body (31). Previous studies have shown that *Forsythia suspensa* extract can improve oxidative damage in broiler breast muscles, and the combination of cinnamon and probiotics can regulate oxidative stress and alleviate kidney toxicity in broilers (32, 33). Therefore, this study explores the effects of AEF and AEF + LP on the antioxidant capacity of goslings under stress conditions. The activity of key antioxidant enzymes in serum, liver, and ileum was measured, and the results showed that adding AEF and AEF + LP to drinking water could increase the levels of CAT, T-SOD, and GSH-PX in serum and reduce MDA levels. Adding AEF and AEF + LP to drinking water could increase the levels of CAT, T-SOD, and GSH-PX in the liver and ileum while reducing MDA levels. In conclusion, adding AEF and AEF + LP to drinking water has antioxidant effects and can enhance the oxidative capacity of goslings. Under stress conditions, Astragalus can increase SOD activity and reduce MDA levels in lactating sows (34). Epimedium has the ability to remove extracellular free radicals, decrease levels of reactive oxygen species, boost the activity of antioxidant enzymes, and improve antioxidant function (35). *Lactobacillus plantarum* improves oxidative damage caused by weaning in young goats, effectively reducing MDA content in the small intestine and increasing duodenal GSH-PX activity (36). This experiment evaluated the expression of antioxidant genes in the duodenum and liver, and AEF and AEF + LP significantly enhanced their antioxidant capacity after stress treatment.

Certain components in the serum can reflect the physiological status of animals under stress. Total protein (TP) in the serum comes from hepatic synthesis and intestinal absorption, with TP mainly comprising albumin (ALB) and globulin (GLB). The more common cause of TP reduction is the decrease in ALB, which is the major protein component of serum TP, playing a role in maintaining plasma osmotic pressure and transporting nutrients (37). GLB is produced by immune organs and has a regulatory role in immune response, anti-inflammation, and antibacterial functions. GLB tends to decrease when the organism is attacked by pathogens. Studies have shown that enzymatic activity and oxidative stress markers, including aspartate aminotransferase (AST) and alanine aminotransferase (ALT), which are important indicators reflecting liver function (38). When the liver is damaged, the levels of ALT and AST significantly increase (39). The concentration variation of serum creatinine (CREA-S) is mainly determined by the glomerular filtration capacity (glomerular filtration rate) of the kidneys. Decreased filtration capacity leads to an elevated concentration of creatinine. Increased blood creatinine levels often signify renal impairment. Studies have demonstrated that Chinese herbal medicine has the ability to significantly promote growth performance, improve blood chemical indicators, exhibit anti-inflammatory qualities, prevent damage, and boost antioxidant capacity (40). Probiotics are commonly employed to enhance blood biochemical parameters in animals. *Lactobacillus acidophilus* is a beneficial bacterium that lives in the intestines, developing a mutually beneficial relationship with the host. On the other hand, *Bacillus subtilis* is a kind of bacterium that may create spores but does not typically reside in the intestines. Supplementing *Lactobacillus acidophilus* and *Bacillus subtilis* in broilers can reduce plasma concentrations of ALT and AST (41). Therefore, this study explores the impact of AEF and AEF + LP on blood biochemical indicators in stressed goslings. The results show that after stress, serum levels of ALT, AST, TC, CREA-S, UREA, CK, TBA, LDH, and P increase, while ALP, TP, ALB, GLB, A/G, and TG levels decrease. The treatment with AEF and AEF + LP induced positive changes in blood biochemical indicators. Studies have shown that astragalus can enhance antioxidant capacity in fish and also has a positive alleviating effect on blood biochemistry (42). Epimedium can improve liver damage caused by the elevation of serum aspartate aminotransferase after oxidative stress (43). Extracts of ligustrum can improve the elevation of lactate dehydrogenase and creatinine in the serum of rats caused by oxidative stress (44). Plant lactobacillus has been confirmed to improve glycerophospholipid metabolism disorder induced by oxidative stress in mice (45).

When the organism is influenced by exogenous or endogenous factors, oxidative stress occurs in the organism. Corticosterone (CORT) is a crucial indicator for assessing oxidative stress. CORT is a glucocorticoid secreted by the adrenal glands, and changes in CORT can reflect the physiological stress of animals. When animals are under stress, CORT levels increase (46). This is consistent with our findings. When the organism is exposed to hydrogen peroxide stress, CORT levels increase, but with AEF and AEF + LP treatment, CORT levels decrease. In a stressed state, the organism activates the hypothalamus-pituitary–adrenal axis, leading to elevated blood glucose levels due to the secretion of glucogenic hormones (glucocorticoids, adrenaline, growth hormone, etc.). Our results are also in line with this pattern, showing a reduction in blood glucose levels with AEF and AEF + LP treatment.

The intestine is the primary organ for nutrient absorption in animals, with nutrients primarily absorbed from the small intestine. The intestinal epithelium is a unique single-layer structure arranged in the intestinal cavity, forming two distinct tissue domains, namely intestinal crypts and villi. Villi are finger-like projections that protrude from the epithelium and lamina propria into the intestinal lumen of the small intestine, serving the function of nutrient absorption. Morphological features of the intestine, such as villus height, crypt depth, and V/C, reflect information about the function and health of the intestinal mucosa. When laying hens are exposed to stressors, the proportion of decreased villus height and crypt depth in the jejunum indicates changes in intestinal morphology (47). In this study, the addition of AEF and AEF + LP to drinking water increased the villus height of the duodenum and jejunum, as well as the V/C of the jejunum. The results indicate that AEF and AEF + LP can restore the villus height of the stressed gosling’s intestinal mucosa. We speculate that AEF and AEF + LP may enhance the proliferation and differentiation of intestinal epithelial cells and the renewal rate of intestinal stem cells. Additionally, AEF and AEF + LP promote the expression of Occludin and TJP1 in the stressed gosling’s jejunum. Astragalus improves the villus length of the Mexican duck’s small intestine and effectively stimulates the mucosal immune barrier, enhancing mucosal immune function (48). Studies suggest that AEF and AEF + LP enhance mucosal integrity, providing a favorable environment for host intestinal health and improving immune resistance to pathogen attacks.

Innate immunity is the first line of defense against the invasion of pathogenic microorganisms in the body. When the body is subjected to external stimuli, it activates a battle mode, increasing the inflammatory response. Inflammatory factors are essential indicators in the process of immune response. When piglets and chickens undergo oxidative stress, it can lead to certain important intestinal diseases and inflammatory reactions (49). Pro-inflammatory factors such as TGF-*β*, TNF-*α*, IL-1β, IL-6 significantly increase during oxidative stress (50). The results indicate that when goslings are subjected to H_2_O_2_ stress, TGF-β, TNF-α, IL-1β, IL-6 are consistent with the above research results. Simultaneously, when AEF and AEF + LP are administered, it can alleviate the inflammatory response. Early weaning can cause intestinal inflammation and diarrhea in animals or humans, and Astragalus has anti-inflammatory activity. They pointed out that Astragalus can improve the expression of IL-6, IL-Iβ, and TNF-α after weaning, and improve the morphology of the jejunum villi (51). *Lactobacillus plantarum*, by optimizing the intestinal barrier, regulating the intestinal microbiota, and inflammatory pathways, reduces IL-1β, TNF-α, and improves the inflammatory response (52).

Poultry has three primary immunological organs: the spleen, thymus, and bursa of Fabricius. The spleen, thymus, and bursa of Fabricius are significant immunological markers that might represent the immune condition of an organism when it experiences a stress reaction (53). The spleen is responsible for filtering pathogens and waste in the blood, clearing old red blood cells, and participating in the regulation of immune reactions in the body. The thymus of poultry plays important roles in immune regulation, metabolic regulation, and developmental regulation. The bursa of Fabricius is the central immune organ in birds, capable of producing B lymphocytes, thereby generating specific antibodies to accomplish specific immune responses. Research indicates that when broilers undergo oxidative stress, the immune organ index and humoral immunity tend to decrease (54). This experiment investigated the impact of AEF and AEF + LP on immune organ indices in goslings under stress. Our findings suggest that immune organ indices decrease when the organism experiences stress, and this condition is alleviated by the administration of AEF and AEF + LP. Additionally, we investigated the effects of H_2_O_2_ stress on the meat quality and color of goslings. In this context, L* represents brightness, a* represents redness, and b* represents yellowness. The results indicate that there is no significant change in L*, a*, and b* when subjected to stress or treated with AEF and AEF + LP. The pH value of the stress group is significantly higher than that of the control group at 45 minutes, and there is a decreasing trend in the pH value of the stress group at 24 hours compared to the control group. Drip loss significantly increases under stress. When treated with AEF and AEF + LP, goslings exhibit a decreasing trend in pH at 45 minutes and an increasing trend at 24 hours, significantly alleviating the exacerbation of drip loss caused by stress and improving meat quality.

The gut microbiota can modulate the immune system, regulate intestinal absorption, thereby improving host health and enhancing productivity, playing a crucial role in livestock and poultry health management (55). Piglets who have been weaned and are under oxidative stress suffer from compromised intestinal function, which in turn causes diarrhea. Consequently, this modifies the composition of the intestinal microbiota and affects the overall growth performance (56). Bacteria belonging to the phylum Firmicutes are mostly Gram-positive bacteria, and they play a crucial role in the host’s nutrition and metabolism through the synthesis of short-chain fatty acids. Through their metabolic byproducts, bacteria of the phylum Firmicutes indirectly connect with other tissues and organs, regulating hunger and satiety. However, when broilers undergo heat stress, the abundance of Firmicutes is significantly reduced (57). Our research indicates that the Firmicutes phylum is the most abundant bacteria in the cecal microbiota of goslings, constituting over 50% of the total bacterial population. Due to the addition of AEF and AEF + LP to drinking water, there is a shift in the relative abundance of the Firmicutes phylum at the phylum level, which is beneficial for gut health. Ruminococcus is a Gram-positive, anaerobic bacterium that appears spherical and does not produce spores. Ruminococcus is one of the earliest discovered bacteria in the stomach, playing a crucial role in metabolism. It has functions such as stabilizing the intestinal barrier, reversing diarrhea, and increasing energy. Our results indicate that, compared to the control group, there is a significant reduction of Ruminococcus at the genus level in the stress group. However, the relative abundance of Ruminococcus is increased when administered with AEF and AEF + LP. Ruminococcus is one of the most efficient bacteria in carbohydrate decomposition, and in the human intestine, it can reduce dietary resistant starch, thereby regulating intestinal homeostasis and improving gut health (58).

This study demonstrates that adding AEF extract and AEF + LP to drinking water can enhance the intestinal health and growth performance of H_2_O_2_-stressed goslings. Treatment with AEF and AEF + LP enhances the intestinal bacterial composition, intestinal morphology, immune response, and growth performance of stressed goslings. Furthermore, AEF and AEF + LP exhibit a greater impact on the immune response, intestinal microbiota, and antioxidant capacity of stressed goslings. AEF and AEF + LP can be used as a health product to improve stress in goslings. This study makes a significant contribution to the sustainable development of animal husbandry.

## Data Availability

The data presented in the study are deposited in the NCBI repository, accession number PRJNA1262860.
